# Understanding the impact of digital therapeutic engagement in promoting mental wellbeing for Pacific youth in Aotearoa New Zealand: an exploration of the literature

**DOI:** 10.1186/s13033-024-00633-x

**Published:** 2024-06-06

**Authors:** Taulaga Auva’a-Alatimu, Siautu Alefaio-Tugia, Julia Ioane

**Affiliations:** Massey University, School of Psychology, Auckland, New Zealand

**Keywords:** Pacific wellbeing, Youth mental health, Digital mental health, Pacific worldview

## Abstract

The Pacific population in Aotearoa New Zealand is youthful, with the majority (55%) being under the age of 25 (Statistics New Zealand, 2014). It is vital that youth mental health for Pacific is understood in relation to their overall wellbeing (Paterson et al., 2018). In parallel to this, the World Health Organization (2022) accentuates the need to protect and promote mental wellbeing for young people globally. Specifically, Pacific youth were far more likely than Aotearoa New Zealand European counterparts to have poorer mental health and higher numbers of suicidality and self-harming behaviours (Ataera-Minster & Trowland, 2018; Fa'alili-Fidow et al., 2016). Moreover, research confirms that Pacific people aged 15–24 years have higher levels of psychological distress of 38% compared to 35% of Pacific adults aged 45–64 years (Ataera-Minster & Trowland, 2018). There is a lack of evidence-based psychological approaches that are culturally appropriate and applicable for Pacific people in Aotearoa New Zealand. Considerably, substantial evidence supports the need to provide more accessible resources and interventions that are flexible, culturally adaptable and cost-effective for Pacific youth. This review aims to (1) provide an insight into Pacific people in Aotearoa New Zealand, (2) have an understanding of Pacific worldview & wellbeing, (3) highlight mental health for Aotearoa New Zealand youth & globally (4) identify therapeutic approaches, including digital mental health globally and in Aotearoa New Zealand.

Understanding the perspectives of Pacific youth is a significant first step. Therefore, this article will examine the therapeutic approaches, specifically in the digital space, that are proven effective when promoting wellness for Pacific youth.

## Introduction

The term ‘Pacific, Pacific Islander, Pasifika, Pasefika, Pacifica’ are collectively used to refer to the diverse cultures from Polynesia, Melanesia, and Micronesia [46]. While the term developed in Aotearoa New Zealand (NZ) prescribes homogeneity, Pacific people are far from this. They are described as a diverse, vibrant, youthful, and thriving population [66].

The seven main Pacific ethnic groups include Samoan, Tongan, Cook Islands, Fijian, Niuean, Tokelauan, and Kiribati, making up 7% of the Aotearoa NZ population [66, 87]. Within the Pacific groups, Samoan is the largest group, contributing to 49%, followed by Cook Island Māori (21%), Tongan (20%), Niuean (8%), and Fijian (5%) (Pasefika [65, 88]). Most Pacific people reside in the North Island, with 66% living in the Auckland region, specifically in South Auckland [87]. It is vital to understand the demographic landscape of Pacific people in Aotearoa NZ, as emphasised through statistics. This demonstrates the history and context that has implications for current and future Pacific generations in Aotearoa NZ.

## Pacific people in Aotearoa NZ

The substantial growth among the Pacific in Aotearoa NZ highlights many benefits and opportunities for future growth and contribution to the economy and society. There has been a gradual increase in those born in Aotearoa NZ, with almost two-thirds of the Pacific population. Hence, there has been a gradual decline of bilingualism (English and one of the Pacific languages) over the last decade [88]. On a positive note, over half of Samoans and Tongans in Aotearoa NZ can hold an everyday conversation in their respective language (Pasefika [65]). Language is immeasurably more than the ability to communicate. The Pacific peoples’ traditional culture, genealogy, and customs are conveyed through the language and oral tradition [92, 102, 104]. The sense of connectedness that language represents among Pacific people is also parallel to the importance of religion/spirituality. Almost 80% of Pacific people are associated with religion compared to 43.8% of the Aotearoa NZ population [89]. Furthermore, Pacific students were four times more likely than Aotearoa NZ European to emphasise the significance of the spirituality [15]. Many studies have proven that religious/spiritual beliefs for Pacific people reinforce a sense of belongingness and connectedness to their family, ancestry, culture, and environment, which ultimately impacts their holistic wellbeing. This is important to note as drawing strength from within the church as faith-based contexts is an area this research seeks to foreground concerning Pacific youth mental health.

## Methods: search strategy

Searches were conducted to gather the literature on the following databases: Scopus, PsychINFO, Medline, Discover, Google Scholar and Web of Science. The keywords used to search the literature focused on this literature review’s aims. The search terms were “youth” OR “young people” OR “adolescence” OR “teen” OR “young adults”, AND “mental wellbeing” OR “psychological wellbeing” OR “wellness” OR “emotional wellbeing” OR “wellbeing” OR “mental health”, AND “Pacific Islanders” OR “Pacific” OR “Pasefika” OR “Pasifika” OR “indigenous”, AND “digital” OR “online” OR “internet” OR “electronic” OR “technology” OR “media.” There was a total of 87 pieces of literature. However, the search was restricted to the period between 2017 and 2022 and confined to only Aotearoa NZ literature, comprising 30 pieces of literature. The existing literature continues to reveal a scarcity and limited evidence related to improving mental wellbeing for Pacific youth by exploring the impact of digital therapeutic engagement. Some themes were highlighted across the searches, which will be explored in this review. A deeper understanding of Pacific youths’ worldview and their concept of wellbeing is a significant step as part of a conversation starter to this literature.

## Pacific worldview: values, relational connectedness, and language

Worldview is defined as a way of understanding one’s perceived world and how they function in it. The ability to make sense of one’s worldview is characterised by core values, beliefs, attitudes, experiences, and constructs [40]. Furthermore, an individual’s worldview affects many aspects of their life. This can be determined by their way of thinking, behaving, and feeling and how they inter-relate with another individual. One worldview is not superior to the other, yet it is vital to consider a person’s worldview to contextualise and understand their perceived way of knowing comprehensively. Being responsive to the needs of one’s worldview is paramount in preventing a biased and judgemental approach that may potentially impact the wellbeing of that individual or group of individuals.

Pacific worldview differs from that of a westernised view. Indigenous psychologies are systems of knowledge and wisdom based on non-Western principles [39]. Pacific people perceive and seek harmony by upholding three critical elements concerning the Pacific worldview of health. The elements are defined by their relationship with Atua (God), Tagata (people), and laufanua (environment/land) [93]. It creates a sacredness among the relationships formed between people, land and spirituality (Te [95]). There is a strong emphasis on preserving and integrating values among the Pacific nations, such as; ‘spiritual, social, physical, economic, and cultural matai (chiefly) systemic values [70], p. 2). Such values are embedded in the Pacific ways of knowing, being and doing and cannot exist without the other. For example, the social structure in the Tongan community is based on a hierarchical system of *kau tu’a* (commoner), *hou’eiki* (nobles), and *ha’a tu’I* (royalty) (Te [95], p. 14). In maintaining these relationships, Tongans have embedded values of *‘ofa* (love, compassion), *faka’apa’apa* (respect) and *fetokoni’aki* (reciprocity and responsibility for each other)” [103], p. 27). As for the Tokelauan culture, māopoopo is the notion of value in which serves as a cultural connector with people and informs fundamental values of etiquette and how one sou.

Pacific worldview is shaped and influenced by Pacific core values. The fundamental values that Samoans uphold include* ‘va tapuia’* (sacred space), *alofa* (love and compassion), *tautua* (reciprocal service), *fa’aaloalo* (respect*), fa’amaualalo* (humility) and *aiga* (family)” (Te [95]. Addressing people’s “physical, mental and social needs” is linked in context to their ‘*olaga fa’aleagaga* (spiritual foundations), *tu ma aganu’u* (customs and traditions), *aiga* (kin and relationship network), and *laufanua* (environment)” [80], p. 42). When considering the entirety of a Samoan individual, it is crucial to preserve and sustain these cultural values, which is at the core of the Samoan way of life *(Fa’aSamoa).* In saying this, how one carries and conducts themselves in their *aiga*, community and environment are vital to the *Fa’asamoa.* A well-known Samoan proverb emphasises that one’s identity and mannerisms are recognised by how ‘one holds oneself, walks and talks.’ In Samoan, it translates to; *‘e iloa le tagata Samoa i lana tu, savali ma lana tautala’* [5], p. 203). In reference to the Tokelauan culture, māopoopo (cultural connector) is a concept that strengthens social interactions and psychologically endows people with a sense of belonging. This is demonstrated through the practice of Te va fealoaki (relationships) between kāiga (families) and extended kāiga [41, 101].

Similarly, ‘the way of being’ for Fijians are defined by expressing *loloma* (love) and *yalo malua* (humility) [64]. The Fijian community also have corresponding core values as other Pacific nations. Implementing and demonstrating such values is paramount when working with Fijian people. These values comprise of; *veidokai* (respect), *veidolei* (reciprocity), *vosota* (patience), *veimaroroi* (protectiveness) and *veivakatorocaketaki* (empowerment) (Farrelly & Nabobo‐Baba, 2014, p. 326). Equally, the Tokelauan’s also uphold values of *loto alofa* (act of kindness), *fakaaloalo* (respect), *loto fehoahoani* (helping others), *loto maualalo* (humility) and *loto fealofani* (united) [41, 101]. Therefore, understanding cultural protocols, customs, and traditions is imperative for Pacific people’s care.

The understanding of self is described as a relational being among Pacific people. Embracing one’s sense of wholeness among Pacific people descends from a place of connectedness within the family, village, genealogy, language, and culture. To disregard this notion among Pacific individuals means a detachment from their sense of self, family, community, and culture. The concept of disconnecting the ‘va’ or sacred space between the individual, family and community is inconceivable for Pacific people [93]. A Pacific perspective of self, others and the world are founded on a collective approach [18].

Furthermore, Alefaio [3] confirms that village life is the epicentre for Samoan people. For instance, Tamasese et al. [93] define ‘self’ as having meaningful relationships with others and not individuals. In other words, viewing the individual as a collective unit is essential, and the identity of Samoans is relational, as stated by Tamasese et al. [93]. Developing relationships is exceptionally important for Pacific people,this requires a deep interpersonal connection (Farrelly & Nabobo‐Baba, 2014; [60]). Maintaining the family and social support connection is critical in promoting mental wellbeing among Aotearoa NZ born Cook Islands [74].

There are fundamental roles and responsibilities that Pacific groups/individuals carry out within societal and familial structures within a cultural context. According to Tamasese et al. [93], honouring space is a Pasifika term for *‘Vā.*’ Understanding the space between people and in-between time is crucial. The symbolism of the space for Pacific people gives meaning and context, and it is ‘not an empty space, nor a space that separates’ [107]. Within *‘vā fealoaloa’i* (relationships of mutual respect), the symbolic space *(va)* that relates and connects one another is *tapu or sa* (sacred). In other words, there is a sense of sacredness in relationships of how one relates to another [93]. The relationship between siblings and parents*,* chief, church minister etc., are defined *tapu* [93]. For example, there is a ‘binding and sacred covenant’ between sister and brother, also known as* ‘Feagaiga’* [100]. Protecting and treasuring the prominence of sisters and women in general are the brothers’ responsibilities and roles to undertake concerning the ‘Feagaiga’ (covenant) [84, 93]. Therefore, it is pivotal to maintain and nurture the space *(tausi le vā)* to avoid breaching sacred relationships *(soli le vā)*. In the achievement of sustaining *vā,* there is an opportunity for one to express themselves in a harmonic, honouring, balanced, and reciprocal manner [55]. Overall, to engage effectively with Pacific people, a need to incorporate a *vā-centred* approach to such relationships is needed.

For many Pacific nations, the cultural traditions, heritages, customs and histories are conveyed through the language or oral traditions [64, 92, 102, 103]. Indigenous knowledge is translated and passed on through oral traditions. Pacific languages are at the core of generating a sense of belonging and identity for Pacific people. Oral traditions for Samoan people are fundamental as this defines their identity and confirms the notion of the *Fa’asamoa* (Samoan way of life) [92]. In addition, the native language among Niueans is highly valued and is linked with a sense of identity and belonging [102]. Upholding the significance of language generates a sense of connectedness, enabling Niueans to express their narratives freely. It is vital to allow Pacific people to share their stories and experiences because it will help contextualise and provide insight into their worldview.

Similarly, Tongans also regard *‘talanoa’* (to talk/communicate) and oratory as sacred, ancient knowledge passed down through generations. The Tongan language has been conveyed and translated through stories, performances and symbols [104]. Creating a safe space *(“vā”)* for Tongans allows them to express and interpret their realities, experiences, and narratives. Overall, oral language for Pacific people is paramount as it connects them to their cultural identity, traditions, and ancestry. Understanding one’s language, engagement modalities, and therapeutic processes among Pacific people will provide meaningful insight into the practicalities and principles of therapy.

## Understanding pacific wellbeing

In recent years, WHO defined mental health as a “state of wellbeing in which the individual realises his or her own abilities, can cope with the normal stresses of life, can work productively and fruitfully, and is able to make a contribution to his or her community [108]. Considering a holistic approach in delivering care to individuals is fundamental and aligns with Pacific people’s perceptions of health and wellness. Pacific cultures perceive mental health to be ‘inseparable from the overall wellbeing of the body, soul and spirit’ [56]. The view of health from a Westernised perspective is holistic, encompassing the physical, mental and social wellbeing and not merely the absence of a disease [44], p. 125). The Pacific concept of health incorporates the spiritual element, also included by WHO [44]. Equivalent to the definition of health by WHO, the *Fonofale* model (Pasifika model of health) was founded by Pulotu-Endermann [73]. This Pasifika model encapsulated the holistic view of health in a Pacific context, using the metaphor of a Samoan *Fale* (house). The four poles of the *Fale* symbolised elements essential to Pacific cultural groups, labelled as physical, spiritual, mental and other (cultural, family, context, time and environment) [73]. Promoting wellness meant that such elements were equally respected. For instance, cultural and spiritual beliefs are equally fundamental as physical health. However, an imbalance on one of the elements could potentially impact the overall wellness of the Pacific individual. There are other Pacific models that also promote and conceptualises mental wellbeing from a Pacific-Indigenous lens, including frameworks such as Te Vaka Atafaga [42], Pandanus Mat [2], and Popao model [23], which encompasses an integrated and holistic approach to wellbeing. The concept of health from a Pacific perspective is vital to understand when delivering care as the individual is approached in a whole and holistic manner. Thus, adopting ‘Pacific ways of knowing’ will enhance Pacific wellbeing by implementing the four elements of the *Fale* to deliver a holistic approach for Pacific people [66, 73].

In contrast to evidence presenting the view that Pacific concepts focus on the collective and holistic approach, an alternative perspective illustrates that eurocentrism or Western research generally embraces an individualistic approach to epistemology. Merry et al. [53] confirm that western psychological models emphasise an individual’s internal psychological state. A few studies have explained how psychology traditionally has been Eurocentric, originating from a White, middle-class value system [35, 38]. According to [35], ‘white culture is the synthesis of ideas, values, and beliefs merged from descendants of White European groups.’ Traditionally, the concept of individualism has been at the core of psychological approaches. Meaning the individual is the primary focus, and their autonomy and independence are highly valued. It highlights that the individual is in control of their environment. The relevance of science and empiricism for Western cultures are fundamental in their ability to make sense of the world they live in [38]. Other components of the white culture clarify that the immediate family is ideal but not at the core. Individuals’ status and power are ‘measured by credentials, positions, and economical possessions’ [35], p. 618).

Western ideas and science concepts are constructed from a Eurocentrism lens [28]. Thus, health was perceived based on the scientific rationality known as the biomedical model in the Western world [33]. Historically, western biomedicine focused on pathophysiologic processes and the cure of diseases and, at times, disregarded the individual’s spiritual, mental, and social wellbeing [61]. While traditionalistic psychological approaches were developed by White clinicians enmeshed in Western cultural values [38]. When using psychological models, there has been a substantial shift in considering other non-Western cultures in modern psychology. With the vast complexities of cultures and populations, there is an emphasis on making psychological theories more responsive to the needs of such groups [38].

The perceptions of health, mental illness and holistic care differ from Western and non-Western worldviews. According to Te Pou [95], the western perspective of mental illness is produced by a ‘chemical imbalance’ in the brain. The Pacific view of mental illness differs, affirming that a spiritual curse or breaches of ‘*tapu/sā’* (sacred) relationships cause mental illness [93],Te [95]. There are formalities and processes in a cultural context that assists in healing and restoring the breaching of sacred bonds and a spiritual curse. Traditional healers and well-respected leaders (religious/family/district) and chiefs (matai) of the community may treat or heal such illnesses [93],Te [95]. There is a need to explore solutions to the challenges associated with culture clashes between a Eurocentric medical model of mental illness and indigenous explanations of mental illness.

Having established different perspectives, stigma related to mental illness contributes to one of the many reasons preventing Pacific people from seeking psychological help [6]. For Pacific people, how they interpret and view mental illness, as mentioned earlier, is a significant step to consider. For Samoans, mental illness is referred to by various terms; *ma’i valea* (mad or stupid), *ma’i o le māfaufau* (brain abnormalities) or *ma’i aitu (*caused by spirit possession) [45], p. 256). Equally, other Pacific cultures share the same interpretation and perspective. For Tongans, *fakasesele* (act silly) and *vale* (foolish, incapable) are phrases used when referring to one having a mental illness, which is interpreted as being mad, insane or crazy [45],Te [95]. Tuvaluans also view those with a mental illness as *fakavalevale,* meaning crazy, linking it with spirit possessions, social wrongdoings, and bizarre behaviours as causal factors [45]. Samu and Suaalii-Sauni [79] explored Pacific perspectives on delivering cultural competencies within mental health. Learning about the different definitions and interpretations of mental illness for Pacific people was essential to consider. This research reported that Cook Island Māori had a derogatory connotation when referring to one suffering from a mental illness,*neneva* (stupid) and *pana’marama* (gone bonkers). They also found that Niueans, similar to other Pacific ethnicities, believed that there was a spiritual possession by a demon or ghost associated with a mental illness, termed *hu aitu*. However, for Fijians, *cavuka* was commonly used in reference to mental illness, signifying being ‘broken’ or ‘snapped’ [79].

According to Ataera-Minster and Trowland [6], the inadequacy of delivering culturally appropriate health models and the lack of incorporating Pacific worldview concepts in mental health raises many concerns, resulting in barriers to access to health care for Pacific people. Fa’alogo-Lilo and Cartwright [16] recently conducted a study to gain a deeper understanding of Pacific peoples' challenges when accessing mental health services and the supports that promoted their engagement. The stigmatisation associated with mental health problems was a significant barrier which resulted in the fear and shame of experiencing mental unwellness from a Pacific perspective. This led to a complex of underlying issues. Some Pacific service users believed they were disappointed, bringing shame to their families by being mentally unwell. Furthermore, the lack of understanding and the mistrust of services contradicts Pacific values and beliefs (Fa’alogo-Lilo & Cartwright, 2021). Significantly, Pacific people tended to utilise services if the providers were more culturally responsive and respectful of Pacific practices. Consequently, when a clinician considers a non-Western culture through Western glasses, there is a potential to neglect an understanding of the non-Western culture since the schemata for remembering them are not proven by their science [39]. That being so, considering and having a deeper understanding of the views of Pacific people is a good starting point in exploring this further. For example, Psychologists could be at the forefront of research that encompasses different worldviews toward universally relevant theory and equality. This would require training to better equip clinicians to work culturally sensitively, reflecting equality and pluralism [38]. Developed countries, such as New Zealand, have imported and utilised Euro-American psychological approaches and principles of the global north among people with underlying mental health issues for decades. However, research suggests that to deliver a holistic care approach specifically for Pacific people, we need to understand and incorporate the Pacific views of health and wellness [6, 17, 66]. Understanding worldview, indigenous psychology, and Pacific ways of knowing needs to be discussed to understand this better [4]. Firstly, there is a need to explore the occurrence and prevalence of mental health among Pacific youth in Aotearoa NZ to determine the relevance of what constitutes good mental wellbeing whilst addressing the challenges present.

## Pacific youth mental health in Aotearoa NZ

Considering their relevance and contribution to today’s society and the leaders of tomorrow, young people's mental health is crucial. Roosevelt [76] summarises the importance of developing youth growth,‘we cannot always build the future for our youth, but we can build our youth for the future.’ Equipping the youth with tools to cope with the world of tomorrow is a significant step. Providing opportunities for young people to grow and learn life skills of dealing with adversities and challenges is fundamental. Improving Pacific youth wellbeing, focusing on developing programmes to enhance resilience and wellbeing skills, is one of the priorities set by the government [57]. In contrast, there seems to be a drastic increase in psychological distress among young people over the last decade (Te [95]). There are many theories and hypotheses associated with such an increase. Some of these reasons include the impact of smartphones and social media, significant childhood events and trauma, socio-economic factors, increases in perfectionism and expectations in schooling/other areas, and future worries concerning employment, adulthood and housing challenges [22], p. 17). Teachers in Aotearoa NZ support incorporating mental health learning into the curriculum to teach and help young people learn how to regulate their emotions and develop resilience and coping strategies when faced with life stressors [66]. This research aims to explore the needs of Pacific youth when promoting mental wellbeing and effectively implementing interventions appropriate and applicable to this community. Thus, the relevance reflecting on Pacific youth data concerning the prevalence of mental health is a good starting point.

According to Fa'alili-Fidow et al. [15], Pacific youth are far more likely to suffer mental disorders than older Pacific people. Research suggests that there are high levels of psychological distress among Pacific people aged 15–24 years (38%) than Pacific adults aged 45–64 years (35%) [6], p. 4). New Zealand’s survey of the health and wellbeing of high school students reported that Pacific students were significantly more likely than Aotearoa NZ European counterparts to have poorer mental health and an increased number of self-harming behaviours [15]. The trend between 2012 and 2019 for mental health among Pacific youth has worsened, resulting in an increase of 25% of depressive symptoms from 14% [22]. The strength of the evidence continues to demonstrate the demand in seeking ways to improve youth mental health, specifically Pacific youth. Furthermore, compared to the rest of the Aotearoa NZ population, Pacific youth alarmingly (specifically young men) have the highest suicide rates [6, 15, 66]. Pacific youth are less likely to access mental health services than the rest of the Aotearoa NZ population [15]. There seems to be a lack of knowledge and understanding of mental illness among Pacific people, thus impacting their engagement with services to help improve their mental health (Fa’alogo-Lilo & Cartwright, 2021). Treatment is unlikely to be effective, or carrying out the appropriate intervention may be compromised due to this misunderstanding. Fleming et al. [19] confirm that services must focus on delivering high-quality digital tools that address critical areas for Pacific wellbeing. There is an emphasis on incorporating the Pacific worldview, cultural identity, spirituality, sense of belonging and connectedness which are essential to Pacific youth in improving mental wellness [57, 66, 74].

Building on the collated evidence, this section illustrates specific data for youth in Aotearoa NZ. The Youth2000 is a series of health and wellbeing surveys including over 36,000 young people from Aotearoa NZ high schools, Kura Kaupapa Māori, alternative education, and teen parent units. The findings from the Youth19 survey indicated an increased rate of depressive symptoms among Pacific females of 33%, compared to 15% of Pacific males [22], p. 10). However, it has been noted earlier that Pacific youth, mostly males have the highest suicide rates in Aotearoa NZ. Tiatia-Seath [97] researched mental health service engagement when implementing suicide prevention strategies for Pacific people. The study emphasised integrating a cultural rather than a clinical approach when applying appropriate engagement methods. There is a need to gain further insight into the reasons for such alarming statistics. Also linked to poor mental health and suicide among Pacific youth is poverty, specifically those living in high deprivation areas. Socioeconomic status significantly impacts the mental wellbeing of individuals. The Youth19 results showed higher depressive symptoms among Pacific students who lived in high deprivation areas (25%) than those in low deprivation areas (15%). Furthermore, a more significant proportion of Pacific students who attempted suicide in the past 12 months lived in highly deprived areas (14%) compared to those in low deprived areas (2%) [22]. In comparison to the general population, Pacific youth continue to report having a higher prevalence of attempting suicide in the past 12 months (12%) than the European (Pakeha) peers (3%) [22]. Concerning statistics and findings from Youth19 highlight an urgency to address the underlying issues. Enhancing mental health services that incorporate a holistic approach that addresses the social, cultural, and economic determinants of wellbeing is fundamental for the Pacific youth [22]. Improving mental health also means fostering a strong cultural identity and belonging among Pacific young people. Working holistically with Pacific people emphasises encompassing culture, spirituality, and family, which has been pointed out earlier. This may provide insight and inform mental health research and practice when delivering such care among the Pacific population. A study carried out by Field Vaka, Holroyd, Neville, and Cammock (2020) illustrated the significance of having such an understanding. The research aimed to capture the perspectives of Tongan youth and mental health service users with mental distress. This study showed that Tongan youth and service users associated mental distress with the biopsychosocial constructions of mental distress. Traditional Tongan views of mental distress resulted from disruptions to social and spiritual relationships and spiritual curses [105]. As for NZ-born Cook Island youth, maintaining social and cultural connections is vital for promoting mental wellbeing [74]. Puna and Tiatia-Seath [74] sought to understand the positive mental wellbeing of NZ-born Cook Islands youth, seeking to work towards implementing strategies for suicide prevention and improving mental wellness. Cultural connections were integral to positive health for NZ-born Cook Islands young people. Such connections were linked to their “social support networks,pride in their Pacific ethnic identities; cultural participation; and language retention” [74], p. 103). A further review led by Tucker-Masters and Tiatia-Seath [99] examined anxiety and depression among Pacific youth who resided in Westernised countries and the Pacific region. The themes of spirituality and religion underpinned mental wellbeing for Pacific youth, along with culture and family [99]. Having a religious affiliation and maintaining a strong sense of spirituality from a Pacific lens is seen as a protective factor for mental illness and promoting wellness [6, 96, 99].

Pacific worldviews are pivotal to how Pacific youth comprehend wellness, especially as these emphasise a holistic understanding. As such, it is essential to note that a burgeoning youth population, poverty, culture, and identity loss altogether exacerbate mental health and cannot continue to be examined in isolation. Mulder et al. [63] echo that those who are in low-income and deprived populations are more prone to developing psychological distress. The socio-cultural factors for Pacific youth contribute tremendously to their holistic wellbeing. It has been highlighted earlier that the traditional psychology approach demonstrated a disconnection between Western and Pacific mental health perspectives. Consequently, understanding the holistic needs of Pacific youth is a priority when delivering psychological treatment [19, 62, 63]. Hence, this research aims to examine Pacific youth's needs when informing and implementing clinical research and practice, ultimately enhancing positive mental wellbeing.

## Youth mental health in Aotearoa NZ

Aotearoa NZ’s youth suicide rates are alarmingly the highest in the OECD and have increased substantially in recent years [66, 71]. Some have described the high rates of suicide as a ‘national shame’ [66], p. 9). The suicide rates are higher for younger people than older people in Aotearoa NZ, particularly males aged 24–44 [66]. The most recent Aotearoa NZ youth survey results indicated that depressive symptoms were more significant among all population groups but significantly higher among Māori, Pacific, and Asian females and increasingly high among rainbow youth and those with disabilities [19, 22]. High levels of psychological distress have drastically impacted young people in NZ; rates have almost doubled from 13% in 2012 to 23% in 2019 [22]. The evidence suggests that psychological distress leads to anxiety, deliberate self-harm, risk-taking and other troubling behaviours among youth [66], p. 49). In particular, students who lived in high deprivation areas and attended low decile schools reported having substantial depressive symptoms [22]. Significantly, seeking help and knowing where to get help is challenging for young people, particularly during distressed and depressed times [6, 22]. For instance, 24% of female students aged 15 and older revealed that they had difficulty getting help during times of distress, compared to 14% of males [22]. The literature supports the notion that young people in Aotearoa NZ are less likely to seek professional support, specifically among indigenous and minority youth [21, 67]. This raises many questions as to why this may be. A study in Aotearoa NZ discovered that one of the many explanations associated with suicidality is the inability to discuss issues of mental distress, resulting in a young person’s incompetence to cope and respond accordingly to stress [31]. There is little evidence of understanding young people’s views on seeking mental health support.

It is devastating that the mental health statistics among the various youth ethnic groups such as Maori, Pacific and Asian youth in Aotearoa NZ have not improved over the recent years [6, 22]. Positive mental health is drastically declining for Aotearoa NZ youth, specifically among Māori and Pacific. Evidence suggests that various circumstances may cause the decline. For instance, the lack of access to mental health services, the impact of socioeconomic inequalities, and cultural differences and perceptions of mental health among the diverse groups in Aotearoa NZ [34, 66]. Fa’alogo-Lilo and Cartwright (2021) accentuates the importance of ongoing training and education for non-Pacific workers in the mental health workforce to promote cultural responsiveness when working with Pacific people, as literature has proven that it is not a one-size-fits-all approach. This requires a greater understanding of Pacific practices, values, spirituality, family and societal structures, alongside other mental health approaches (Fa’alogo-Lilo & Cartwright, 2021).

## Global indigenous youth mental health

Elevated levels of depression and anxiety are not only confined and unique to Aotearoa NZ. Nevertheless, international findings have also mirrored similar patterns in North America, England, Australia and other places in the world [11]. According to Polanczyk et al. [69], there is a high prevalence of mental health issues among youth and children worldwide, and it has soared considerably over recent years. Therefore, the severity of mental health problems can heighten the perceived likelihood of suicide. Globally, the second leading cause of death for ages 15–29 is suicide [110]. The literature reveals consistent findings about the prevalence of mental health issues among the younger population. For instance, in the UK, 50% experience mental distress by the age of 14 and 75% by 24 [10]. There is a global urgency to address and implement appropriate interventions and strategies to prevent the alarming statistics. Internationally and nationally, indigenous and minority young people are at greater risk of developing mental health issues. In Hawaii, the Native Hawaiian youth report having a higher prevalence of suicide behaviours than non-Hawaiian youth [113]. Mental health problems among the younger population have been extensively researched, some studies have proven a greater prevalence of mental health issues among minority groups, and others reported lower or similar rates [90]. Pacific people in the United States were confirmed to have higher rates of mental illness than non-Pacific people, stating 4.8% of Pacific people suffered depression compared to 1.5% of American Asians and 3% of the total population [1]. More specifically, youth mental health among those living in the Tokelau Islands has shown an increase in suicide rates over a decade ago [94]. A study by Tavite and Tavite [94] pointed out several contributing factors that increased the risk of suicidal behaviours among Tokelauan youth, including breakdown in social relationships, unresolved grief, academic failure and behaviours that brought about shame and humiliation.

Furthermore, the native indigenous youth (15–19 years) of Australia, known as the Aboriginals and Torres Strait Islander peoples, identified a severe mental illness of 31.6%, compared to non-Aboriginal or Torres Strait Islander youth 22.2% [7]. Significantly, many of the young people from minority groups around the world are less likely to seek mental health support than Europeans and are unlikely to receive appropriate care [6, 58, 72, 75, 99]. For example, Chavira et al. [12] found that Latino children were not accessing mental health services due to significant barriers. These include language difficulties, logistic factors, beliefs about causes, stigma linked to mental health treatment and social support (p. 54). Additional contributing factors to the lack of access for indigenous youth in Australia are fear, intergenerational stigma, language differences, shame, and the inadequacy of appropriate services [72].

The global insights mentioned seem to parallel some of the challenges that Pacific young people battle with [6],Fa’alogo-Lilo & Cartwright, 2021; [99] Clearly, there is a demand to improve mental health services that cater to the needs of young people. One therapeutic approach that has demonstrated effectiveness in improving youth wellbeing globally and locally is digital mental health [19, 24, 53]. Considering the evidence from the literature, it is paramount that therapies and mental health interventions provide a holistic approach, incorporating social and cultural concepts when delivering care for minority groups over-represented in Aotearoa NZ and internationally.

## Therapeutic approaches including digital mental health globally

There has been substantial research on the effectiveness of Cognitive behavioural therapy (CBT) as a form of psychological treatment to undertake and manage mental health problems internationally and nationally [9, 43, 91],Te [95]. However, seeking psychological treatment can be challenging for young people. Some of the reasons are the cost of therapy, stigmatisation linked to mental distress, long waiting lists, and the demand for services that exceed the availability of clinicians [6, 66]. One approach proven to improve mental health and wellbeing among youth is digital mental health tools (DMHTs) [19, 24, 53, 68]. To pursue mental health support for low- and middle-income countries are becoming affordable and feasible globally, and the younger population have shown to engage more with digital technologies [49, 59, 77]. Recent studies imply that digital mental health approaches (including online, computerised, smartphone, and app-based), can be as efficacious and acceptable as traditional face-to-face therapy [24, 71]. The numerous benefits of DMHTs are highly cost-effective, hugely accessible, and the ability to deliver high fidelity [53, 71]. This has a vast potential to reach a large population of young people worldwide, for instance, millions worldwide download mental health or wellbeing apps annually [19], p. 2). If developed appropriately, digital mental health interventions can be easily scalable and modifiable [19].

A recent report called Aotearoa NZ Digital Tools for Mental health and Wellbeing outlined various methods of DMHTs which range from short and brief interventions to extensive clinical therapies [19]. Websites provide opportunities to educate and inform people with mental health information, especially those taking an initial step in seeking mental wellbeing support [14, 19, 109],Young [112]. Self-help tools are digitally designed to offer a brief intervention or complete computerised therapy without needing support from a mental health professional. A brief intervention delivers a short and specific approach, such as a breathing exercise [19]. There has been much evidence supporting computerised therapies which delivered a CBT approach over a specified period. This approach can either be guided or used as a self-help [13, 78, 86]. Guided help also consists of having therapy support with a clinician via telephone (telehealth), web chat or online [19]. Apps are sources of digital tools, intended to offer a brief intervention to promote and improve mental wellness. Similarly, chatbots are automated chat agents that deliver therapy using chat functions [19]. The evidence highlights that DMHTs are worth considering. In particular, a study conducted by Kayrouz et al. [36] aimed to examine the effectiveness of a self-guided and modified internet-delivered CBT (iCBT) version of the Arabic Wellbeing Course among Arabs with depressive and anxiety symptoms aged 18 and over. The online course included five lessons offered for eight weeks. Thirty-six participants were selected from seven countries around the world. The participants reported a significant improvement, with an overall reduction in depressive, anxiety and psychological distress symptoms [36]. Further research in Vietnam explored youth’s perspective on mobile phone-based health-related interventions [98]. Participants selected were 356 youths (aged between 15 and 25 years). The findings indicated low usage of mobile health-related apps among the youth. There was also a lack of apps in the Vietnamese language, which may have caused issues in understanding the content and navigation of the apps. Most participants were not aware of web-based health interventions. However, using digital mental health tools was appealing and 54 percent reported that it was ‘integral for the mobile apps to have a sharing/social network functionality’ [85, 98], p. 7).

Mental health apps are most used among youth in promoting mental wellbeing. However, there seems to be a gap in the literature assessing the end users’ needs in utilising the app tools. Kenny et al. [37] conducted a study that targeted adolescents from Ireland and interviewed 34 participants aged 15–16 years. The study aimed to examine their needs and concerns regarding mental health app tools and provide feedback using a mental health app prototype named ‘CopeSmart’ [37]. The findings revealed positive feedback concerning the mental health app prototype, recommending that this may be acceptable to adolescents. There were common themes that participants voiced regarding mental health apps. The importance of safety and ensuring that their information was confidential. Another critical point to consider was to ensure that apps were relevant, engaging, and functional. Lastly, participants expressed that apps must be accessible, user-friendly, affordable, or free of charge [37]. To elaborate further on the effective use of mental health apps, Headspace is one of the most-used apps for depression and anxiety [19, 37]. The app also provides a digital approach to youth aged 12–25, offering free, confidential service with support from a mental health professional via phone, chat, or email. Headspace was initially designed for Australian adolescents and their friends and families across Australia [29]. Like headspace, the MeeTwo app or website serves the same purpose and function. The MeeTwo app is well received among young people in the UK. There is approximately 6000 youth who use it monthly [51]. Users have found the app helpful in improving wellbeing and self-esteem [51]. On the other hand, some young people conveyed their interest in a DMHT with a gaming approach. A problem-solving game based was created called “Problems, Options, Do it (POD)” [25]. A pilot study led by Gonsalves et al. [25] aimed to investigate the feasibility and acceptability of the POD app delivered via a smartphone in Indian secondary schools. Participants were selected from two secondary schools in Goa, India. A total of 230 participants completed the intervention. The results revealed significant improvements on all measures at 12 weeks. The participants had a positive experience using the app. The simplicity of the intervention made engagement easy, ultimately improving the mental wellbeing of the students [25].

In contrast to the evidence presented, it is critical to note the limitations of using DMHTs. For instance, a recent review reported that although there are 10,000 mental health apps, only 10% have been clinically or empirically tested [50]. Whilst there are emerging DMHTs and research available, significant challenges remain. The lack of access to digital technology is problematic for youth in low-income groups. For instance, having sufficient finances to access a device, internet connections and electricity when charging the device’s battery can be challenging [27, 32, 49]. An underlying barrier to utilising DMHTs for youth is the lack of awareness of DMHTs and the uncertainty of accessing relevant information [85]. The disadvantage of delivering DMHTs is not having the maximum potential to reach the targeted audience. Evidence suggests that many people seek mental health support until they become severely distressed [19]. Therefore, future research needs to prioritise and improve access to digital interventions to impact on a larger scale the younger population. Unfortunately, the app retention rate is 4% of users at three months, and 25% only use an app once [8]. The retention rate and poor uptake of DMHTs are worth exploring, especially for Pacific youth given that there is little evidence in the literature. Fleming et al. [19] have provided some solutions to enhance reach and access for young people. Some solutions include increasing promotion/endorsements, rising diversification of tools and integrating digital tools into community health promotion activities and the social services [19], p. 23). Increasing awareness about DMHTs among adolescents is crucial and encouraging the youth to use the digital platforms they are already familiar with to promote health messages is relevant and ideal. For instance, Facebook, Twitter, TikTok, WhatsApp, television and radio [29, 37].

## Therapeutic approaches including digital mental health for youth in Aotearoa NZ

Table 1 (Appendix) has been created to collate and summarise the literature related to DMHTs available in Aotearoa NZ.

There are many different approaches that young people engage effectively in when seeking mental support. In Aotearoa NZ, it was discovered that younger people were three times more likely to use DMHTs than seeing a family doctor and four times more likely to utilise a phone line when seeking psychological support [22]. Among the different ethnic groups in Aotearoa NZ, each has their own needs and preferences when seeking help in times of distress. According to [21], some youth in South Auckland reported that they mainly talk to family friends and post on social media when they feel distressed rather than seek mental support online. However, for some Pacific people, accessing online interventions can be a positive due to avoiding the disclosure of personal issues in the family, as this can potentially bring about the embarrassment and breach the sacred relational space *(soli le vā)* between family members [19, 104].

A study was carried out in 2012, which aimed to explore some Aotearoa NZ youths’ perspectives on depression, help-seeking and computerised therapy [20]. Out of the 39 participants, 15 participants were of Pacific descent who aged between 13 and 16 years. The results showed that some were hesitant to discuss mental health issues with clinicians; however, there was a great interest in computerised interventions helping with depression. Similarly to international literature, the participants of this study admitted that the barriers to accessing computerised therapy might be not having access to devices and the shame and stigma associated with mental illness [20].

Several studies successfully instigated the development of the well-known DMHT in Aotearoa NZ. SPARX (also known as Smart, Positive, Active, Realistic, X-factor thoughts) is a well-known e-therapy tool for young people aged 12–19 who suffer from depression or anxiety [52]. SPARX has been empirically supported with evidenced-based research globally and locally, targeting Aotearoa NZ young people [13, 52, 83, 111]. Furthermore, the SPARX programme is a free online computer game that provides psychoeducational tools and CBT skills to treat depressive and anxiety symptoms [53].

Merry et al. [53] researched the effectiveness of the SPARX tool among Aotearoa NZ youth. The research design was a randomised controlled non-inferiority trial, and 187 adolescents (aged 12–19) participated in the study. Out of the 187 participants, 15 participants were of Pacific descent. The SPARX group consisted of seven modules over four to seven weeks, and the treatment group received face-to-face counselling by a trained clinician. The findings revealed that there was a decrease in depressive symptoms of those in SPARX (10.32) compared to the treatment group (7.59) [53]. Moreover, remission rates were more significant in SPARX (43.7%) than in treatment (26.4%) [53]. This study proved that SPARX is a potential alternative for treating youth with depressive symptoms in primary care settings and has the capability to deliver some of the challenges faced in seeking treatment [53]. Furthermore, a computerised psychological approach can contribute positively to Aotearoa NZ youth’s mental wellbeing. Although this study provided some significant findings, there is still minimal knowledge of the applicability and the effective use of SPARX specifically among Pacific youth considering the small sample of participants of Pacific descent who participated in the study. Exploring Pacific youth’s perspective on the utilisation of SPARX and the cultural relevance and responsiveness of this intervention tool could be investigated.

Those DMHTs that Māori researchers and communities have created also aim to promote mental wellbeing among *Rangatahi* or young people. Shepherd et al. [82] interviewed 26 Māori indigenous youth and their families to explore their views on using a prototype computerised CBT (cCBT) program named SPARX. The discussions raised were linked to the game's outline and structure and culture's significance. The results showed positive feedback, reporting ‘good face validity for Māori indigenous youth and viewed it as an appealing and culturally relevant programme [82].

Evidenced-based literature supports therapeutic interventions that make cultural identity the focal point in establishing relationships and connections to promote Māori wellbeing [106]. A programme that has complemented a holistic and cultural worldview of health is the Ol@-Or@ app, developed with Māori and Pacific communities to help prevent the risk of developing non-communicable diseases (NCDs) [54]. The Ol@-Or@ is a mobile health (mHealth) programme that targets indigenous populations (Māori & Pacific) over 18 years old who may be at risk of developing NCDs. The app incorporated a culturally tailored approach to healthy eating, physical activity, decreasing stress, improving sleep, and weight management [54]. Māori and Pacific's worldviews were considered when carrying out health activities, emphasising spirituality, cultural concepts/customs, proverbs, and culturally tailored motivational messages.

The findings showed an overall improvement in adherence to health-related behaviours over time. However, there were no significant differences between the intervention and control groups [54]. Overall, there was a low engagement with the intervention among the participants. Issues concerning low engagement may have led to barriers to data access, adequate storage on phones and digital literacy level [54]. This finding is also parallel to the international literature discussed earlier.

Despite the programme not being youth-specific, Ol@-Or@ is the first research to co-design and evaluate a mHealth intervention for Māori and Pacific who are at risk of NCDs [54]. Therefore, the digital intervention will inform and provide insight for future strategies and policies relating to the health and wellbeing of Māori and Pacific people.

Whilst there is a dearth of literature related to therapeutic approaches, specifically DMHTs among indigenous youth, research still supports the importance of centralising the needs and demands of the younger generation when considering psychological approaches [19, 54, 77]. For instance, many Māori youth use social media sites and apps because many online social platforms encourage a sense of connectedness while maintaining whanau (family) relationships, which is pivotal for the wellbeing [26]. Some literature suggested that social media platforms should be integrated when designing Māori interventions as Māori people are already familiar with them. Also, whanau and human connections are fundamental among Māori and Pacific people [6, 82].

LeVa organisation is a Pacific mental health and addiction service working closely with the Pasifika community. LeVa supports Pacific families and individuals with mental health and addiction problems and offers other services, such as suicide, violence and primary prevention [48]. Mental Wealth Project (MWP) is an online mental health literacy education programme for Pasifika youth, established by LeVa [48]. The online intervention aimed to provide knowledge, education and support relating to mental health for Pasifika individuals and their families. Workshops are also encouraged in which LeVa offers face-to-face seminars in schools, sports clubs, churches and community settings [34, 48]. Other Pacific digital tools that LeVa has implemented consist of; the *Atu Mai website* (Violence prevention), *Aunty Dee app* (problem-solving app), *Flo: Pasifika for life website* (suicide prevention), *Mana Restore website/app* (gamer wellbeing) and many other educational tools [48]. Although there is no evaluative information available yet on the Aunty Dee app, several clinicians have reviewed this app on the Health Navigator New Zealand website, which should be considered alongside the other sources of evidenced-based DMHTs. One psychologist praised the Aunty Dee tool, detailing how fun and interactive the tool was for youth, specifically for Pacific youth (Health Navigator New [30]). Another clinician explained how beneficial this tool would be for youth and applicable for a diverse audience. The review highlighted the importance of assisting the app user in recognising their problems by weighing out the pros and cons to identify potential solutions. A further reviewer affirmed how relevant this tool was for those struggling with a lot of complex or varying issues and has the potential to help people navigate and find ways to manage their own life stressors (Health Navigator New [30]). Some users of the Aunty Dee app reported that they would recommend this to a friend stating that it was simple to use and provided good tips and examples of how to manage their current problems in an organised and helpful way (LeVa, 2016b). One reviewer specified that Aunty Dee could be further enhanced by incorporating an audio version with the opportunity to record verbal responses from the users. (Health Navigator New [30]).

Many Pacific DMHTs have been utilised among young people in Aotearoa NZ, precisely Pacific people [48, 54]. However, there is a lack of empirically supported literature on the effectiveness of such tools among Pacific youth. Thus, it generates a gap in research and the need to prioritise the development of therapeutic approaches among Pacific youth is paramount. There are substantial limitations of access, reach, and retention with DMHTs mentioned earlier, and this research explores the potential of whether DHMTs are impactful when promoting Pacific youth's mental wellbeing.

Numerous DMHTs emphasise the cognitive and emotional aspects, with a minimal focus on other elements of wellbeing, notably spirituality [19]. Māori and Pacific's worldviews need to be embedded in the future development of therapeutic approaches to promote mental wellness. No research has examined the effectiveness of a brief online CBT intervention for Pacific youth in Aotearoa NZ. Furthermore, there is a lack of evidence-based psychological approaches that are culturally appropriate and applicable to Pacific people in Aotearoa NZ.

Recent research proved that DMHTs that delivered an individual approach in their intervention were ineffective and contributed to the low levels of engagement and non-completion of the programme [19, 54, 77]. On the other hand, therapeutic approaches that provided increased human-to-human support by local communities and Pacific providers were more likely to engage in the digital intervention [19, 54]. Parallel to this finding, Ataera-Minster and Trowland [6] reported that 52% of Pacific participants voiced that they would first seek support from a friend or family member if they or someone they knew suffered from depression or anxiety. In saying so, some Pacific respondents were unaware of DMHTs or online sources [6]. Recently, Pacific youth in the Youth19 Brief views reported on the significance of being offered options for human contact and support during times of distress (King-Finau, 2022). Moreover, Pacific youth wanted digital tools to be simple and user-friendly. Speaking to family or friends was stressed, including via online tools and incorporating pathways with human connection when using digital tools (King-Finau, 2022).

Engaging in apps for CBT may not necessarily be appealing to youth, specifically to Pacific youth [19]. For instance, Pacific participants reported lower digital use than others. Similar to a study mentioned previously, Pacific participants expressed that they would prefer to talk with a family member, friend or someone in their community if they were distressed (85%) compared to using digital mental health tools (8%) [19]. Some of the findings in the report by Fleming et al. [19] highlighted the need to develop digital tools that incorporated Pacific core values that were parallel with Pacific world views, specifically meeting their needs and wants. This potentially may be more appealing and relevant for Pacific people to engage with.

Being equipped with tools, and guidance from clinicians and social connections, are the therapeutic processes that empower and promote positive mental wellbeing for Pacific people. Knowing where to promote health information is crucial for Pacific youth. The literature supports that using social media platforms to post health and wellbeing messages (Facebook, Instagram, TikTok and websites) is familiar and well known among Pacific communities [19],King-Finau, 2022; [54, 77]. Evidently, Pacific youth confirmed that they wanted wellbeing online sources to be more accessible and easier to find. Removing barriers to accessing digital tools is key. So implementing wellbeing messages on platforms that already exist and are used by Pacific young people has the potential to improve engagement (King-Finau, 2022). Working collaboratively with community and church leaders is essential when exploring ways to engage effectively with Pacific communities [6, 48, 66]. Significantly, the desire to strengthen networks in the Pacific community and work closely with those supporting Pacific youth may be a way to improve mental wellness. More research needs to position Pacific worldview and values at the core when developing DMHTs better to inform theory and clinical practice within mental health.

## Future implications

There is an assumption that Pacific young people will use online/e-therapy tools because they are young and are more familiar with digital technology. However, based on practice experience of the first author with Samoan young people, this does not appear to be the case. Pacific youth engaged in a Pasifika mental-health prevention programme developed by the first author offered different experiences and opinions regarding DHMTs. For example, Pacific youth engaging with digital technology used this as a means of social interaction/connection and a forum to express their thoughts/feelings/opinions. Youth reportedly engage with various social media platforms excessively, such as Facebook, Instagram, Tik Tok, and Snap Chat (social media) as opposed to app-based technology. Some youth members at our local church used the Bible App during the Covid lockdown period to strengthen and empower their mental wellbeing. They used this app to connect and invite church and family members to share verses and daily devotions for spiritual encouragement. This highlights different cultural beliefs, practices and worldviews that shape Pacific youths’ perspectives (as detailed throughout this article) and impact their engagement and utilisation of e-therapies. As such, from practice experience encountered, it is clear that engaging in DMHT’s will be different for Pacific youth and as a gap in research and literature, this is an area that needs further exploration. Whilst social media platforms are used a lot by Pacific youth for various reasons, seeking professional help may not intentionally be at the forefront. Promoting mental wellbeing may be perceived differently from the lens of a young Pacific person. Ways of increasing self-care activities and healthy behaviours can be actioned in various forms. For instance, connecting with a peer digitally, learning a routine dance on TikTok, reading a motivational post, watching a peer go live, and so on. It may not be an approved or appropriate e-therapy or psychological app. However, it may serve the same purpose in promoting mental wellness for Pacific youth.

## Conclusion/recommendations

Research suggests that the attempt to provide wellbeing messaging where young people already are is crucial [19]. The key insight from this research was that understanding and valuing Pacific youths’ needs in engaging with therapeutic modalities such as DMHTs are vital for providing culturally relevant care. The need to incorporate Pacific worldviews and values into DMHTs aims to inform clinical practice and theory in mental health. There is an increasing awareness that providing culturally relevant and appropriate care to Pacific people living in New Zealand requires a deeper understanding of valuing the realities and unique experiences they bring, both collectively and individually [6, 57].

Conclusively, this scoping exercise reviewing research and literature examining Pacific youth's mental health needs in relation to DMHTs has found a gaping hole in what is available. The gaps in this research are compounded by the scarcity of DMHTs that are culturally appropriate for non-European communities, specifically Pacific people. A further gap in research requires further exploration and evaluation of studies on DMHTs and its efficacy of use in Aotearoa NZ. To date, there has been no published research on psychological approaches, integrating spirituality and a digital intervention for Pacific youth in promoting mental wellbeing, despite health policies of Aotearoa NZ emphasising the need to strengthen Pacific people knowledge and skills to promote autonomy related to their wellness [57, 66]. Existing interventions developed in Aotearoa NZ are used by Pacific youth (LeVA tools, SPARX and mental health apps). However, there is limited empirical evidence to support that these tools are effective for Pacific youth. A better understanding of Pacific youth’s impact and perspectives on existing DMHTs will highlight the potential benefits, limitations and gaps related to the engagement of such tools. Considering recent studies by Fleming et al. [19], (King-Finau, 2022), it is paramount to develop therapeutic approaches that promote mental wellbeing via social media platforms as this is a preferred option for Pacific youth when it comes to engaging digitally as opposed to utilising DMHTs [19]. The therapeutic approaches that are appropriate and applicable for Pacific youth are at the centre of this understanding and exploration. Furthermore, delivering a clinical cultural intervention approach is a significant step that can enhance Pacific youth engagement and ultimately contribute positively to their mental wellbeing.

## Data Availability

All data generated or analysed during this study are included in this published article (and its supplementary information files).

## Appendix

See Table 1.

**Table 1:** An overview of digital mental-health tools, specifically in Aotearoa NZ

**Aotearoa**					
[20]	This study was developed to contribute to under- standing the needs and preferences for depression interventions among young people alienated from mainstream education and to consider their views on computerised interventions and c-CBT in particular	The views of young people alienated from mainstream education on depression, help seeking and computerised therapy	13-16-year-olds	Focus groups with 39 young people in alternative schooling programmes for students alienated from mainstream education	The use of computer programmes that are appealing and can be provided in non-stigmatising ways appears to be one promising approach
[53]	To evaluate whether a new computerised cognitive behavioural therapy intervention (SPARX, Smart, Positive, Active, Realistic, X-factor thoughts) could reduce depressive symptoms in help seeking adolescents as much or more than treatment as usual	Computerised gamified cognitive behavioural therapy (CBT). (SPARX) comprising seven modules delivered over a period of between four and seven weeks, versus treatment as usual comprising primarily face to face counselling delivered by trained counsellors and clinical psychologists	12-19-year-olds	94 participants were allocated to SPARX and 93 participants to treatment as usual. 38% of the participants were of Pacific descent	Showed that participants who played SPARX had a higher reduction in depression than the control group. The same was found at a 5 months follow up
[81]	To conduct semi structured interviews with Māori youth (taitamariki) and their families to explore their perspectives on a prototype computerized cognitive behavioural therapy (cCBT) program called SPARX	To explore the relevance of SPARX to Māori, including the cultural acceptability of designs and content, and of the perceived relevance of SPARX to Maori	16-18-year-old adolescents; (taitamariki), “taitamarikis’ mothers, and family (whanau)	26 participants were involved. There were 7 focus groups on the subject of the design and cultural relevance of SPARX that were held	Māori participants were positive about the SPARX prototype and considered it both appealing and applicable to them. Cultural relevance was viewed as being important for the engagement of Māori young people with SPARX and Whanau are important for Māori youths’ wellbeing
[54]	Aotearoa Māori and Pacific- focused app (OL@-OR@) that mainly targets physical activity and physical health ([19]	The OL@-OR@ trial was a 12-week, two-arm, cluster-randomised controlled trial The intervention group received the OL@-OR@ mHealth programme (smartphone app and website). The control group received a control version of the app that only collected baseline and outcome data	18 years or older, had regular access to a mobile device or computer, and had regular internet access	337 Māori participants from 19 clusters and 389 Pasifika participants from 18 clusters (*n* = 726 participants) in the intervention group 320 Māori participants from 15 clusters and 405 Pasifika participants from 17 clusters (*n* = 725 participants) in the control group	There were no significant differences between the intervention and control groups in any secondary outcome. The OL@-OR@ mobile health programme did not improve adherence to health-related behaviour guidelines amongst Māori and Pasifika individuals
(LeVa, 2016a)	**‘Aunty Dee’** wellbeing tool is to help people cope with stressful life experiences through support with problem solving	Aunty Dee provides structured problem solving in a self-reflective way	Pacific and Māori young people aged 14–25 years but Aunty Dee is free for all	Not specified	Micro-intervention features: Quick, interactive problem solving
[47]	Targets Pacific youth and gamers	Website with a game- like look and brief, clear information about mental health and wellbeing topics including sleep and gaming		Not specified	Includes some Pacific focused content
	Mental health literacy skills. Positively framed resources and information to inform and support individuals and others’ re: mental health	Mental wealth resources: Topics include sleep, mindfulness, eating well, alcohol, stigma, depression and anxiety	Pacific youth, adults and families	Not specified	Not specified
